# Multi-agent reinforcement learning with approximate model learning for competitive games

**DOI:** 10.1371/journal.pone.0222215

**Published:** 2019-09-11

**Authors:** Young Joon Park, Yoon Sang Cho, Seoung Bum Kim

**Affiliations:** School of Industrial Management Engineering, Korea University, Seoul, Republic of Korea; Massachusetts Institute of Technology, UNITED STATES

## Abstract

We propose a method for learning multi-agent policies to compete against multiple opponents. The method consists of recurrent neural network-based actor-critic networks and deterministic policy gradients that promote cooperation between agents by communication. The learning process does not require access to opponents’ parameters or observations because the agents are trained separately from the opponents. The actor networks enable the agents to communicate using forward and backward paths while the critic network helps to train the actors by delivering them gradient signals based on their contribution to the global reward. Moreover, to address nonstationarity due to the evolving of other agents, we propose approximate model learning using auxiliary prediction networks for modeling the state transitions, reward function, and opponent behavior. In the test phase, we use competitive multi-agent environments to demonstrate by comparison the usefulness and superiority of the proposed method in terms of learning efficiency and goal achievements. The comparison results show that the proposed method outperforms the alternatives.

## Introduction

Recently, multi-agent reinforcement learning has garnered attention by addressing many challenges, including autonomous vehicles [1], network packet delivery [2], distributed logistics [3], multiple robot control [4], and multiplayer games [5, 6]. Due to the recent progress in deep reinforcement learning that allows the study of many agents in various environments, multi-agent reinforcement learning has thrived. However, most of the recent work considers fully cooperative tasks and communication within agents [7, 8], yet multi-agent competition is one of the crucial domains for multi-agent reinforcement learning. This task aims to coevolve two or more agents which interact with each other in the same environment. Competitive multi-agent reinforcement learning was behind the recent success of Go without human knowledge [9]. Furthermore, the competitive multi-agent environment provides agents with a customized curriculum to facilitate efficient learning and avoid local optimum [10].

In multi-agent settings, including competitive tasks, the problem of reinforcement learning is notoriously complex because two or more agents share an environment. When agents are trained by simple single-agent learning methods, they violate the basic assumption of reinforcement learning that the environment should be stationary and Markovian. For example, one agent’s state depends not only on its own behavior but also on the behaviors of other agents, such as teammates or opponents [11, 12]. In such cases, current state-of-the-art learning methods often fail because the other agent is simply treated as a static part of the environment without considering its learning process. The centralized training of a decentralized policies paradigm can alleviate the challenge of non-Markovian and nonstationary environments during learning [12] and has recently attracted attention in the multi-agent reinforcement learning community [12, 13, 14, 15].

Although the advent of centralized training of decentralized policies can moderate the nonstationary environment problem, learning algorithms still suffer from instability because of the inherent nonstationarity. In particular, it is more severe in imperfect information games whose agents partially observe different parts of states than in perfect information games [16]. Therefore, some research has been conducted to address this problem using opponent modeling [11, 17]. Opponent modeling is based on reasoning about other agents’ intentions and being able to predict their behavior. It is inspired by the human mental process of simulating others’ behavior by interacting with them, which allows humans to understand others’ intentions and act accordingly in social settings. It is also associated with model-based reinforcement learning because it models the policies of opponent agents while considering them as part of the environment. The previous studies demonstrate that opponent modeling leads to better performance than pure model-free reinforcement learning algorithms.

Combined with the opponent modeling, model-free reinforcement learning has shown promising performance for competitive tasks. However, most of the previous studies have relied on fully competitive one-on-one competition tasks [10, 18, 19, 20]. In this study, we focus on many against many competition tasks that contain both cooperation tasks within a team and competitions between opponents. In particular, we consider environments in which agents only observe their team rewards being shared equally by each agent. To handle this problem, algorithms should promote learning through competition with opponents as well as cooperation by communication with teammates. Moreover, multi-agent credit assignment should be considered in estimating the contribution of each agent.

In this study, we propose an actor-critic method for learning policies in a competitive multi-agent environment based on the framework of centralized training and decentralized policies. To learn multiple policies capable of communication, we propose using recurrent neural network-based actor-critic networks with deterministic policy gradients. Because the proposed method allows for the communication and centralized training of the decentralized policies by the gradient flows in recurrent actor-critic networks, it can avoid violating the stationary and Markovian assumptions. Moreover, to enhance the stability of the learning process, we propose approximate model learning for perceiving environment dynamics and opponent behaviors. By using the auxiliary prediction network, approximate model learning can be readily adapted to the existing model-free methods. Consequently, the agents can be well trained under nonstationary environments and partial observability.

The main contributions of this study can be summarized as follows:

1. We propose a competitive training framework using recurrent layers to handle cooperative multi-agent tasks without access to opponents’ parameters. With deterministic policy gradients, agents learn differentiable communication protocols for coordinating with others. In addition, the recurrent critic network helps actors train based on the global reward for each team while addressing the multi-agent credit assignment.
2. We propose approximate model learning using auxiliary prediction networks (AMLAPNs) to address the problem of the partial observability and variance increase of the learned policy. The AMLAPN can estimate approximate state transitions, the reward function and opponents’ behavior to improve the robustness of the learning process. Moreover, the auxiliary prediction network can straightforwardly be combined with model-free reinforcement learning methods without any assumptions about the environment.
3. To demonstrate the usefulness and applicability of the proposed method, we use simulated environments and compare the proposed method with existing methods in terms of learning efficiency and achieving the goal in the test phase. The results show that the proposed method is promising.

The remainder of this paper is organized as follows. The next section presents related works. The third section describes the problem definition and background of the research. The 4^th^ section presents the details of the proposed method, and the 5^th^ Section presents a simulation study to examine the performance of the proposed method and compare it with other methods under various scenarios. Finally, Section 6^th^ contains our concluding remarks.

## Related works

In some multi-agent systems, single-agent reinforcement learning methods can be directly applied with minor modifications [21]. One of the simplest approaches is to independently train each agent to maximize their individual reward while treating other agents as part of the environment [6, 22]. However, this approach violates the basic assumption of reinforcement learning that the environment should be stationary and Markovian. In many cases, the environment of any single agent is dynamic and nonstationary due to the changing policies of other agents [12]. Another basic approach designs multiple agents as a single agent whose action space is the joint action space of all the agents [23]. While allowing coordinated behaviors across agents, this approach is not scalable because the size of the action space increases exponentially with the number of agents. In this study, we tackled these limitations by centralizing the training of decentralization policies.

An alternative is to use model-based policy optimization for learning optimal policies through backpropagation. However, because this approach requires a differentiable model of the dynamics and assumptions of the interactions between agents, it leads to a high computational cost for the optimization process [24]. The proposed method is a model-free reinforcement learning method, making its optimization process efficient.

From a game theory perspective, Heinrich & Silver studied fictitious competitive training for achieving an approximate Nash equilibrium in imperfect information games such as poker [16]. Recently, Foerster et al. introduced an algorithm of which learning rule includes an additional term that accounts for the impact of one agent’s policy on the anticipated parameter update of the other agents, and showed that it can achieve cooperation [17]. However, it only considers one-to-one competition tasks and the algorithm requires access to the opponent’s parameters. Lowe et al. proposed the multi-agent deep deterministic policy gradients (MADDPG) with a central critic that can observe the joint state and actions of all agents to reduce variance [14]. The MADDPG is demonstrated on various many-to-many competition tasks. However, it is hard to apply imperfect information games such as the StarCraft because its learning process must know opponents’ parameters. In this study, we do not assume that the learning algorithm has access to the opponent’s parameters or observations. Moreover, we test fully competitive settings between two adversarial teams in a 2D world with simulated physics.

Opponent modeling has been extensively studied in imperfect information games. Most existing approaches fall into the category of explicit modeling, in which a supervised learning model is built to directly predict the opponent’s action [25, 26], private information [27, 28], or domain-specific strategies [29, 30]. However, most previous approaches have focused on developing models with domain-specific probabilistic priors or strategy parametrizations. In contrast, we propose a general end-to-end framework for opponent modeling using neural networks the does not require any domain knowledge. Davidson applied neural networks to opponent modeling that trained a simple artificial neural network as a classifier to predict opponent actions [31]. Lockett et al. proposed an neural network architecture to identify the type of opponent. However, instead of learning hidden representations, they use a mixture of weights over a pre-specified set of opponents [32]. In addition, Lockett et al. used the neural network as a standalone solver without a reinforcement learning setting, which may be unsuitable for more complex problems. He et al. proposed the deep reinforce opponent network based on deep Q-network to discover strategy patterns of opponents [11]. However, it only considers one-to-one competition tasks. To address limitations of existing studies, we consider predicting opponents’ actions for many-to-many competition tasks. In addition, the opponents’ behavior can be considered as part of environment dynamics in terms of one agent. In this study, we also consider state transition and reward function as well as the opponent modeling. Although inferring environment change surrounding one agents is main idea of the model-based reinforcement learning, we apply this idea to model-free methods without any assumption of the environment.

## Preliminaries

In this study, we consider a competitive multi-agent task with multiple agents that can be described as a multi-agent extension of the Markov decision processes (MDPs) called “partially observable Markov games (G).” It is defined by the tuple G = 〈*S*,*U*,*P*,*r*,*Z*,*O*,*n*,*γ*〉, in which *n* agents identified by a∈A≡{1,…,n} choose sequential actions. *s*∈*S* describes the true state of the environment. Each agent receives a private observation correlated to *s*. At each time step, each agent simultaneously chooses an action *u*^*a*^∈*U*, forming a joint action ***u***∈***U***≡*U*^*n*^. Agents on the same team share the same reward function r(s,u):S×U→R. *γ*∈[0,1] is a discount factor that determines the extent to which the policy favors immediate rewards over long-term gains [33, 34].

We consider a partially observable scenario in which each agent draws individual observations *z*∈*Z* according to the observation O(s,a):*S*×*A*→*Z*. Each agent has an action-observation history τ^a^∈*T*≡(Z×U)*, on which it bases a stochastic policy *π*^*a*^(*u*^*a*^|τ^*a*^):*T*×*U*→[0,1]. The joint policy *π* has a joint action-value function: $Q^{\pi }(s_{t},u_{t})=E_{s_{t+1:\infty },u_{t+1:\infty }}[R_{t}|s_{t},u_{t}]$ where $R_{t}=\sum_{i=0}^{\infty }\gamma^{i}r_{t+i}$ is the discounted return. The agents aim to learn a policy that maximizes their expected discounted returns.

## Proposed method

We consider the problem of multiple agents acting in competitive environments to maximize shared rewards. Therefore, learning communication among agents is important for sharing the information required to handle the tasks. In this section, our goal is to derive an algorithm that works well under the following four constraints: (1) the learned policies can only use their own observations during the execution phase, (2) agents can understand the environment dynamics based on model-free methods, (3) cooperation is possible without sharing information with other agents, and (4) the learning algorithm does not require accessing the opponent’s parameters or observations. The above considerations will lead to a multi-agent learning algorithm that can be applied to the competitive tasks of two adversarial teams. These tasks consist of cooperation within the same team and competition between the two different teams.

### Competitive training framework using recurrent layers (CTRL)

To enable competition between agents of the different teams, we propose a competitive training framework using recurrent layers (CTRL). In this framework, the multi-agent policies of one team are modeled by the recurrent neural network to communicate with each other. Thus, each team can be isolated by each recurrent neural network. Adversarial teams compete with each other in environments while maximizing the rewards shared among teammates. This enables isolated training of each team because it does not require access to the opponent’s parameters or observations. These settings are especially useful for learning in imperfect information games.

To foster cooperation within agents of the same team, we propose a multi-agent deterministic policy gradient with recurrent layers. The proposed method is a combination of centralized training and decentralized execution based on differentiable inter-agent learning. It is thus possible to generate end-to-end learning across agents through a differentiable communication channel in recurrent layers. These settings can share parameters and deliver gradients from one agent to the next using the communication channel.

**Fig 1:**
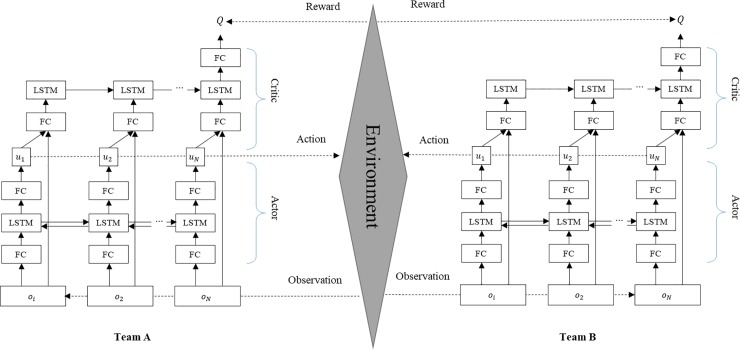
Overview of the proposed CTRL when two adversarial teams exist. Each team is trained independently.

In this study, both the actor and critic networks use long short-term memory (LSTM) layers. In particular, actors consist of bidirectional LSTM layers to reduce the dependency on agent sequences. In the execution phase, the actor network receives a sequence composed of agents’ observations and generates actions for each individual agent. In the training phase, critic networks estimate the Q-value, which evaluates the current states with a sequence of observations and the actions of all agents. The input sequence containing the information of the agents is accumulated sequentially through the recurrent layer. Because recurrent layers can serve as a communication channel as well as a local memory saver, each individual agent can maintain its own internal state and share information with its collaborators. Fig 1 shows the overall architecture of the proposed method.

More precisely, we consider actor networks, i.e., the policy *π*, parameterized by *θ* for the agents of one team. Given N agents, let ***u*** = {*u*_1_,…,*u*_*N*_} be the set of all agent actions and ***o*** = {*o*_1_,…,*o*_*N*_} the set of all agent observations. We can then write the gradient of the expected return for agent *i*, J(θ)=E[R] as:

$$\nabla_{\theta }J(\theta )=E_{s∼\rho ,u∼\pi (s)}[\nabla_{\theta }\log\;\pi_{\theta }(u|o)Q^{\pi }(o,u)],$$ (1)

where *Q*^*π*^ is a centralized action-value function, i.e., critic networks, that takes as input the actions and the observed state information of all agents. It produces a single Q-value that considers the interactions between all agents and an environment. We can extend the above framework to work with the following deterministic policy gradients and the Gumbel-Softmax distribution scheme:

$$\nabla_{\theta }J(\theta )=E_{u,o∼D}[\nabla_{\theta }\pi_{\theta }(u|o)\nabla_{u}Q^{\pi }(o,u)],$$ (2)

where *D* is the replay memory containing the tuples (***o***,***u***,*r*,***o***′), storing the experiences of all agents. According to the above formula, the policy is learned via a gradient on the Q-value. Therefore, if the critic estimates the action-value function satisfactorily, it delivers the appropriate gradient signals to each agent although the agents can only observe a shared reward. The centralized action-value function, i.e., critic, *Q*^*π*^ is optimized by minimizing the following temporal-difference loss:

$$L(\theta_{c})=E_{o,u,r,o\prime }[{\left(Q(o,u|\theta_{c})-y^{DQN}\right)}^{2}],$$

$$y^{DQN}=r+\gamma \;\max_{u\prime }\bar{Q}(o^{\prime },\;u^{\prime }|\theta_{c}^{-}),$$ (3)

where $\bar{Q}$ is the target Q-value function. $\theta_{C}^{-}$ are the parameters of a target critic network that is frozen for a number of iterations while updating the online critic network. The proposed CTRL training algorithm is summarized as follows:

**Algorithm 1**: **CTRL**

**1.**K: number of teams (1,…,*k*,…,*K*)

**2.**Actor and critic networks are parameterized by $\theta_{a_{k}}$ and $\theta_{c_{k}}$, respectively.

**3.**Initialize replay butter, *D*_*k*_

**4.for** episode = 1 to num episodes **do**

**5.**    Receive initial state ***o*** = (*o*_1_,*o*_2_,…,*o*_*N*_)

**6.    for** *t* = 1 to steps per episode **do**

**7.        for** *k* = 1,…, K **do**

**8.**            For each agent *i*, select the action $u_{i}^{k}\approx \pi_{i}^{k}(o_{i})$

**9.**            Execute the action $u^{k}=(u_{1}^{k},u_{2}^{k},\ldots ,u_{N}^{k})$

**10.**            Observe the reward *r* and new observations ***o***′ = (*o*′_1_,*o*′_2_,…,*o*′_*N*_)

**11.**            Store (***o***,***u***,*r*,***o***′) in a replay buffer *D*_*k*_

**12.          *o*←*o*′**

**13.**          // Update

**14.          if** *t* % train interval = 0 **do**

**15.**                Sample a random mini-batch *B*←*m*×(***o***^*j*^,***u***^*j*^,*r*^*j*^,***o***′^*j*^) from *D*_*k*_

**16.**                Compute target values for each sample using a target network

**17.**                        $y^{j}=r^{j}+\gamma \bar{Q}(o^{\prime j},u^{j})$

**18.**                Update the critic by minimizing the loss:

**19.**                    $L(\theta_{c_{k}})=\frac{1}{S}\sum_{j}{\left(y^{j}-Q^{\pi^{k}}(o^{j},(\pi_{1}^{k}(o_{1}),\ldots ,\pi_{N}^{k}(o_{N})));\theta_{c_{k}}\right)}^{2}$

**20.**                Update the actor using the sampled policy gradient:

**21.**                    $\nabla_{\theta_{a_{k}}}J(\pi_{\theta_{a}})=\frac{1}{M}\sum_{j=1}^{M}\sum_{i=1}^{N}\frac{\partial Q^{\pi^{k}}(o^{j},(\pi_{1}^{k}(o_{1}),\ldots ,\pi_{N}^{k}(o_{N})))}{\partial u_{i}^{k}}\frac{\partial \pi_{i}^{k}(o_{i})}{\partial \theta_{a_{k}}}$

**22.        end for**

**23.    end for**

**24.**    Update the target network parameters:

**25.    for** *k* = 1,…, K **do**

**26.**        ${\theta \prime }_{a_{k}}\leftarrow \tau \theta_{a_{k}}+(1-\tau ){\theta \prime }_{a_{k}}$

**27.**        ${\theta \prime }_{c_{k}}\leftarrow \tau \theta_{c_{k}}+(1-\tau ){\theta \prime }_{c_{k}}$

**28.    end for**

**29. end for**

### Approximate model learning using auxiliary prediction networks (AMLAPN)

**Fig 2:**
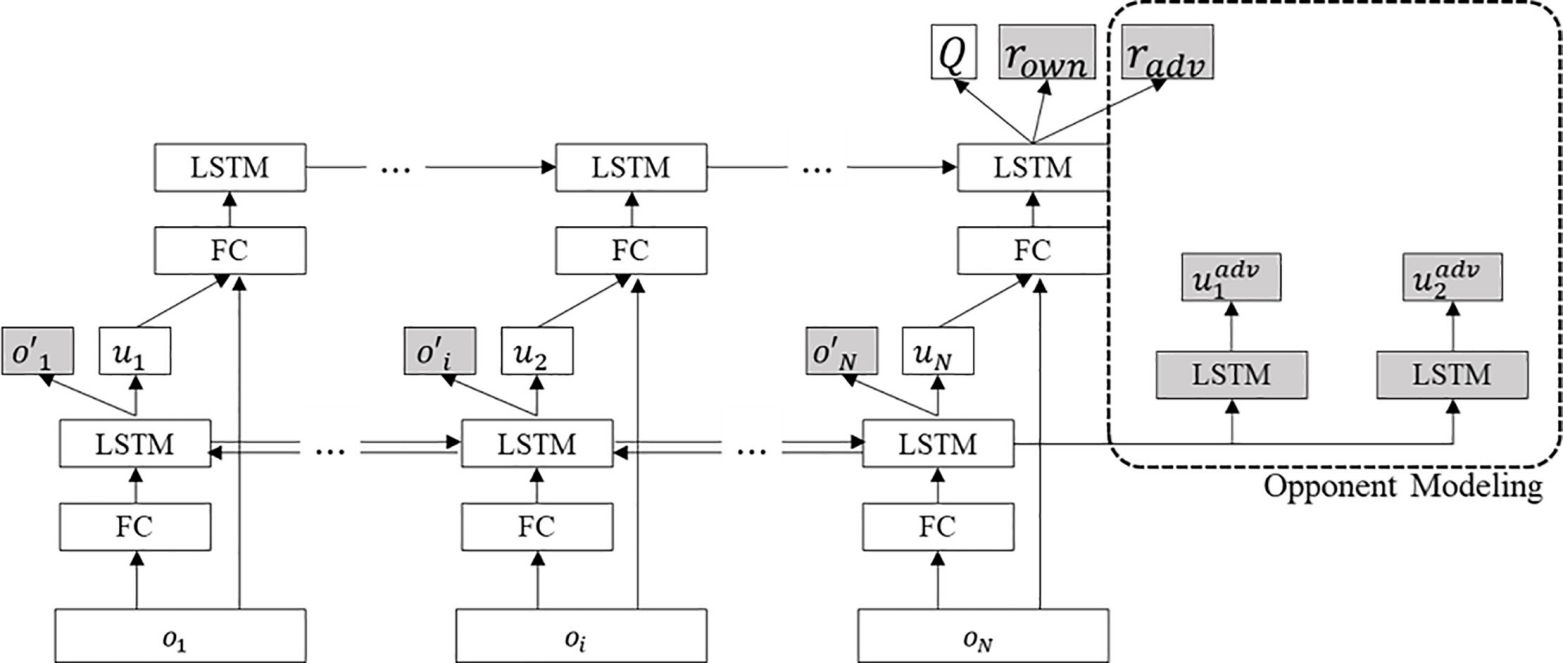
Architectures of the CTRL with an AMLAPN. In the training phase, the observations are sequentially processed by the actor and critic along the arrows. The gradient signals are propagated in the reverse direction of the arrows. The shaded regions represent the auxiliary prediction networks for the approximate model learning. The number of units are shown within parentheses.

To deal with the fact that reinforcement learning algorithms are prone to suffer local optima, we propose AMLAPNs. Learning environment dynamics can be helpful in avoiding this problem. Especially, in competitive multi-agent tasks, environment dynamics depend on not only the intrinsic state transition mechanism but also on an opponent’s behavior. To perceive the dynamics, we consider two auxiliary prediction tasks: Task (1) models environment dynamics for each team, and Task (2) models environment dynamics for adversary teams. Task (1) consists of two supervised learning problems: predicting the next states and rewards based on the observed state transitions. These predictive tasks are linked to model-based reinforcement learning algorithms aiming for the optimal policy by exploiting the dynamics of the environment (i.e., the state transitions). Task (2) can further be divided into two supervised learning problems: predicting the opponents’ policies and rewards based on the information observed. These predictive tasks are an extension of approximate model learning for competitive multi-agent settings. They can stabilize the learning process in imperfect information games by inferring the opponent’s behavior. Although model-based algorithms have the advantage of learning a robust policy and sampling efficiency, they are computationally more complex than model-free methods. Thus, we attempt to embed the advantages of model-based algorithms in our model-free algorithm. Fig 2 shows actor and critic network architectures combined with the AMLAPN.

For learning environment dynamics for each team, Task (1), we proposed the auxiliary next states the prediction networks ($f_{a}^{own}$) residing within the actor networks to promote an understanding of the ways in which agent actions affect the environment. The auxiliary networks predict the next observations of all agents based on their current observations and actions. Furthermore, the auxiliary reward prediction networks ($f_{c}^{own}$) belonging to the critic networks perceive the reward mechanism inherent to the environment associated with the states and actions of all agents. The auxiliary reward prediction networks estimate the reward using the current observations and actions of all agents. Therefore, the actor and critic networks learn the dynamics of the environments more readily than pure model-free learning algorithms. The auxiliary networks on the actor and critic network can be learned by minimizing the following mean squared losses:

$$L_{state}=E_{u,o∼D}\left[{\left({\hat{o}}^{\prime }-o^{\prime }\right)}^{2}\right],\;\mathrm{where}\;{\hat{o}}^{\prime }=f_{a}^{own}\left(o,u\right).$$ (4)

$$L_{r_{own}}=E_{u,o,r∼D}\left[{\left({\hat{r}}_{own}-r_{own}\right)}^{2}\right],\;\mathrm{where}\;{\hat{r}}_{own}=f_{c}^{own}(o,u).$$ (5)

The predicted observations and rewards are not used as inputs when determining actions. They are only used for the purpose of the auxiliary prediction of the observations and rewards which in turn promotes the cognition of complex environment operations.

For task (2), the auxiliary opponents’ actions prediction networks ($f_{a}^{adv}$) are combined with the actor networks to promote the inferring of adversarial agents’ actions. The auxiliary networks predict the actions of all opponents based on their observations. To handle the varying number of adversarial agents, we used LSTM layers. Furthermore, the auxiliary reward prediction networks ($f_{c}^{adv}$) belong to the critic networks to perceive the reward mechanism inherent to the environment associated with the states and actions of all opponent’s agents. The auxiliary reward prediction networks estimate the reward using the current observations and actions of agents. Therefore, the actor and critic networks learn the opponents’ behavior more readily than pure model-free learning algorithms. The auxiliary networks on the actor and critic network can be learned by minimizing the following losses:

$$L_{op}=E_{u,o∼D}[{\hat{u}}_{adv}log\;{\hat{u}}_{adv}],\;where\;{\hat{u}}_{adv}=f_{a}^{adv}(o).$$ (6)

$$L_{r_{adv}}=E_{u,o,r∼D}[{\left({\hat{r}}_{adv}-r_{adv}\right)}^{2}],\;\mathrm{where}\;{\hat{r}}_{adv}=f_{c}^{adv}(o,u).$$ (7)

The outcomes of two auxiliary networks are only used for promoting the cognition of opponents’ behaviors, which are a part of competitive multi-agent environments.

The auxiliary prediction networks are simultaneously trained with policy learning. The gradients augmented by model-based learning can be expressed as follows:

$$\nabla_{\theta_{a}}J(\theta_{a})=E_{u,o∼D}[{\nabla_{\theta }}_{a}\pi_{\theta_{a}}(u|o)\nabla_{u}Q^{\pi }(o,u)-\nabla_{f_{a}^{own}}L_{state}-\nabla_{f_{a}^{adv}}L_{op}],$$ (8)

$$\nabla_{\theta_{c}}L(\theta_{c})=E_{u,o∼D}[(r+\gamma Q^{\pi }(o,u)-Q^{\pi }(o,u))\nabla_{u}Q^{\pi }(o,u)-\nabla_{f_{c}^{own}}L_{r_{own}}-\nabla_{f_{c}^{adv}}L_{r_{adv}}].$$ (9)

Here $\nabla_{\theta_{a}}J(\theta_{a})$ is a gradient for actor networks, and $\nabla_{\theta_{c}}L(\theta_{c})$ is a gradient for critic networks. These gradient signals implicitly contribute to determining actors’ actions while considering state transition and opponent policies. To ensure against environment dynamics and opponent evolution information being forgotten, we maintain an experience replay memory and cull batches of samples in memory at every model update.

## Experiments

### Environments

**Fig 3:**
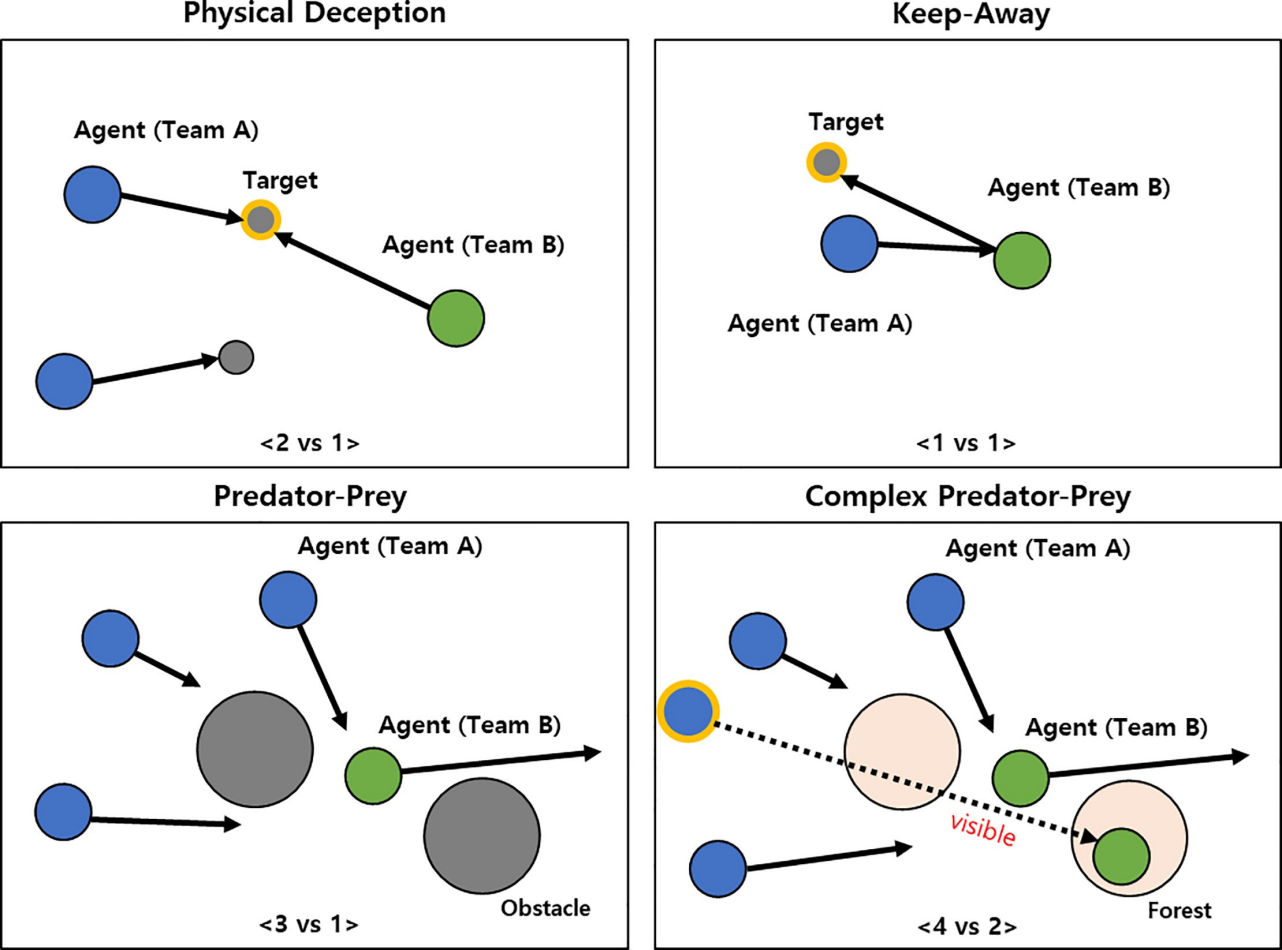
**Illustrations of the experimental environment for four scenarios:** physical deception (*top left*), keep-away (*top right*), predator-prey (*bottom left*), and complex predator-prey (*bottom right*).

We used a multi-agent particle environment to evaluate the capabilities of the proposed method. The environment consisted of two adversarial teams, *N* agents for team A, *M* agents for team B, and *L* landmarks. Teams A and B have different tasks, and they are hostile relations. The environment possesses the properties of two-dimensional continuous space and discrete time, and agents can take physical actions and observe the relative positions of other agents and landmarks based on their own position. Physical actions allow agents to move in the environment. To further simplify the control problem, we used five-dimensional discrete action spaces, enabling agents to move up, down, left, right, or to stay still. However, agents could not move in strict accordance with the given discrete physical action because the framework includes a basic engine that takes into account their momentum [14]. In our experiments, we considered four competitive multi-agent scenarios in which all agents should maximize team rewards. Some scenarios required communication between agents to achieve the best reward, while others required agents to perform physical actions. Fig 3 shows the graphical descriptions of experimental environments of all scenarios.

#### Physical deception

This scenario consists of two agents belonging to team A. They cooperate to reach a single target landmark from two possible landmarks. They are rewarded based on the minimum distance of an agent to the target. However, a single adversary (team B) also wishes to reach the target landmark; the catch is that the adversary does not know which of the landmarks is the correct one. Thus, the cooperating agents, who are penalized based on the adversary’s distance to the target, learn to spread out and cover all landmarks to deceive the adversary.

#### Keep-away

This scenario consists of two landmarks, including a target landmark, two cooperating agents (team A) who know the target landmark and are rewarded based on their distance to the target, and one adversarial agent (team B) who must prevent the cooperating agents from reaching the target. The adversary accomplishes this by physically pushing the agents away from the landmark, temporarily occupying it. While the adversaries are also rewarded based on their distance to the target landmark, they do not know the correct target; this must be inferred from the movements of the agents.

#### Predator-prey

In this variant of the classic predator-prey game, three cooperating agents of team A are the predators and one adversary of team B is the prey. Because the prey is faster than the predators, the agents of team A must, while cooperating, chase the prey around a randomly generated environment with two large landmarks impeding the way. Each time the cooperative agents collide with an adversary, the agents are rewarded while the adversary is penalized. Agents observe the relative positions and velocities of other agents as well as the positions of the landmarks.

#### Complex predator-prey

This scenario is a complex version of the predator-prey game. The overall settings are the same as in the predator-prey game except for forests and foods. Two preys (team A) can hide in the forests to avoid being seen from outside. Four predators (team B) chase the preys. The adversary leader can see the agents at all times and has the responsibility of sending messages to the other adversaries to help coordinate the chase. The prey should obtain as much food as possible while avoiding collisions.

## Results

We examined the performance of the proposed methods under the four scenarios in terms of episode rewards in training and test phases. We compared three proposed methods while varying the levels of approximate model learning. The first method is CTRL without AMLAPN, the second method is CTRL with AMLAPN to enable agents to understand environment dynamics (AMLAPN(own)), and the last method is CTRL with AMLAPN to enable agents to understand environment dynamics and opponent behavior (AMLAPN(adv)). From the first to the last method, the levels of approximate model learning increase. We hypothesized that the AMLAPNs would show better performance because they can perceive the intrinsic mechanism in the environments, including the adversarial team’s policies. In addition, we compared them with the MADDPG, of which learning process although requires accessing the opponents’ parameters. However, the proposed methods assume that the parameters of adversarial agents are unknown.

In our experiments, all methods were implemented such that their number of layers and hidden dimensions or learning parameters were equivalent to those in our method. We used three-layer neural networks with rectified linear unit activations to represent the actor and critic networks. Each network was trained with ten random seeds, and all methods were restricted to experience 40,000 episodes. The size of the replay buffer was one million, and we updated the network parameters after every hundred samples added to the replay buffer. Therefore, all training was conducted using the same amount of experience. In addition, we used an Adam optimizer with a learning rate of 0.01 and *τ* = 0.001 to update the target networks. The discount factor γ was set to 0.95, and a batch size of 1,024 was used. The source code of the proposed method can be found here at https://github.com/yjpark1/competitiveMARL. We implemented the MADDPG using git repositories (github.com/openai/maddpg) written by the authors of the MADDPG papers [14].

**Fig 4:**
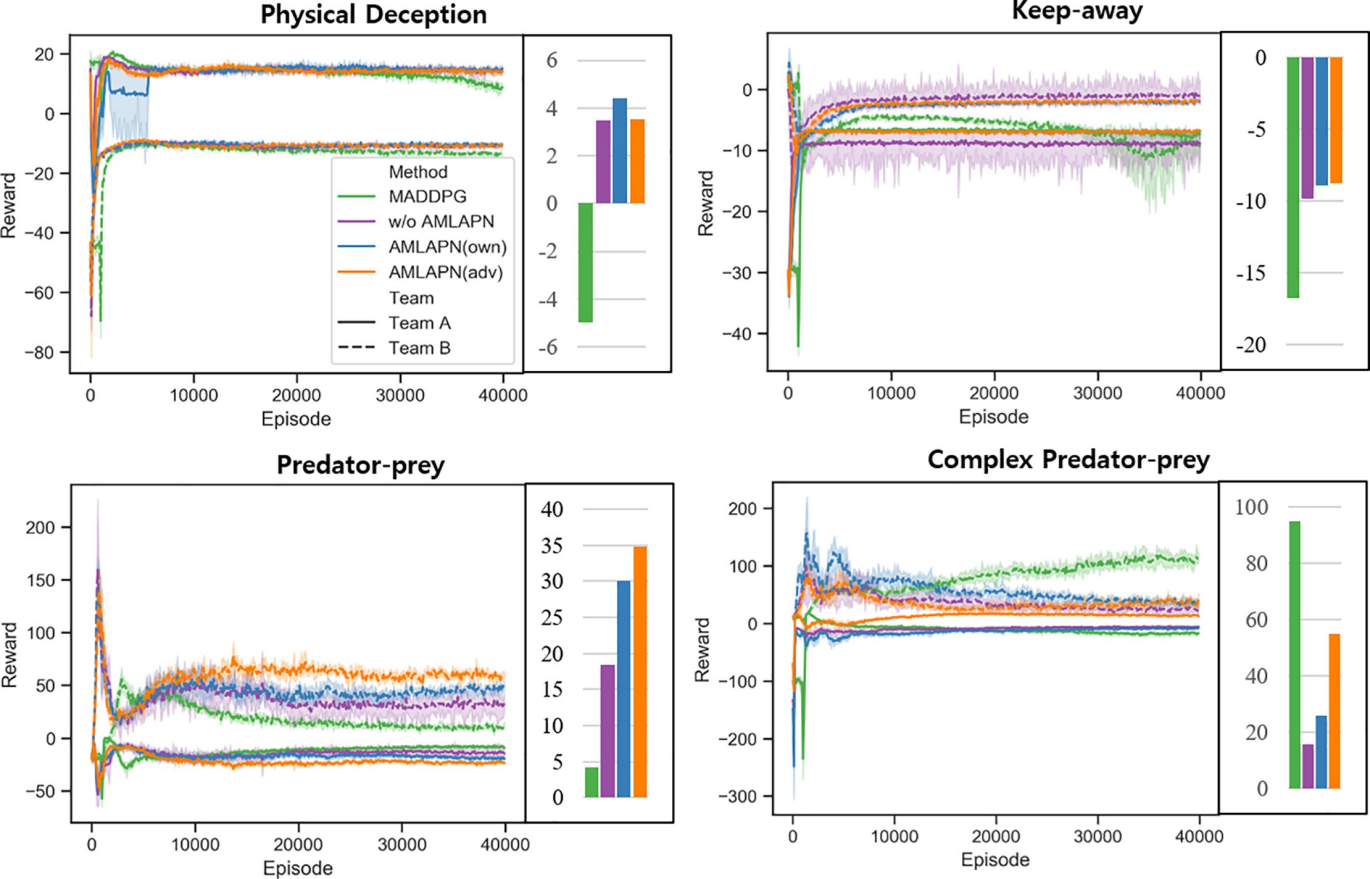
**Learning curves for the four competitive scenarios:** episode rewards in physical deception (*top left*), episode rewards in keep away (*top right*), episode rewards in predator-prey (*bottom left*), and episode rewards in complex predator-prey (*bottom right*) scenarios. Each bar cluster represents the converged episode reward at the end of training. The shading region is a 95% confidence interval across the different random seeds.

Fig 4 shows the learning curves and convergence points of the rewards sum of two teams. Although all methods show similar learning stabilities, each method converged differently in terms of the rewards sum. In most scenarios, the proposed methods outperformed the MADDPG. In particular, the performances of the proposed CTRL demonstrate its effectiveness in learning multiple policies to compete with others. In addition, we could identify that the AMLAPN increased the learning performance of the CTRL. In “keep away” and “predator-prey” scenarios, the convergence of rewards is high; the level of approximate model learning increases while it outperforms the MADDPG. Although the MADDPG is still more effective than the proposed method in the complex “predator-prey” scenario, the CTRL is highly enhanced by the AMLAPN. Thus, we could conclude that the proposed AMLAPN promotes competitive training by perceiving environment dynamics and opponent behavior.

**Fig 5:**
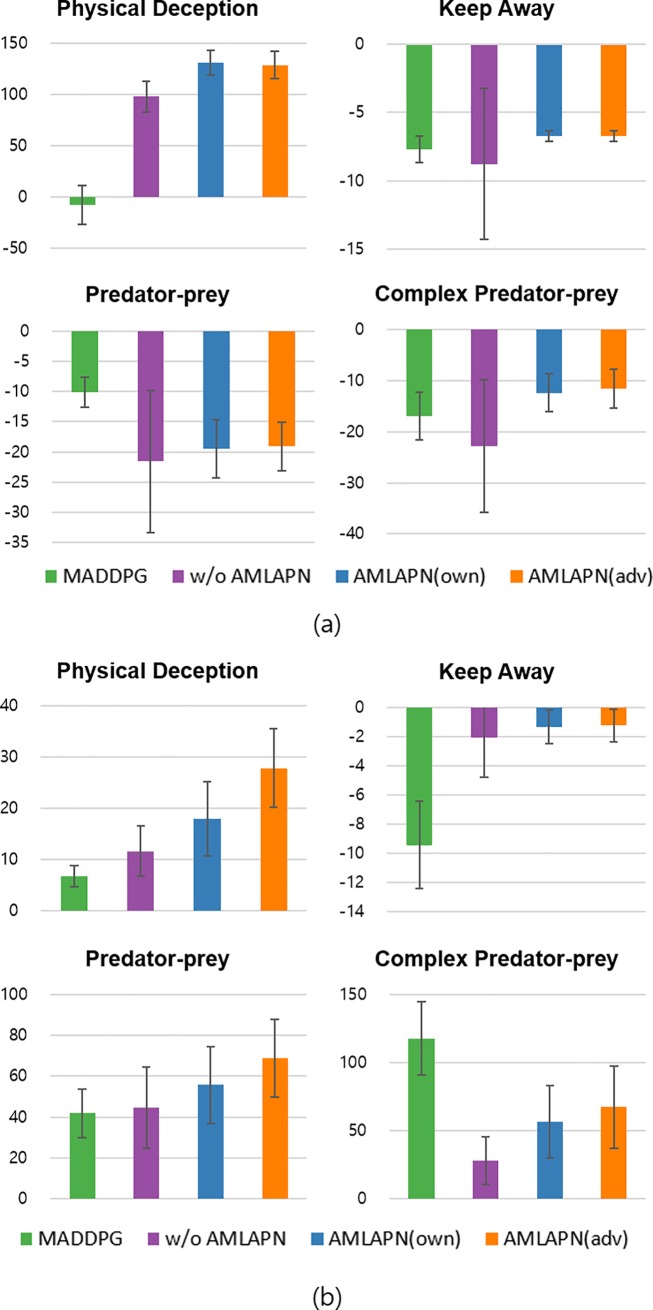
**Relative performances in round-robin tournament evaluations:** the performances of team A trained by four methods (a), and the performances of team B trained by four methods (b). Each bar cluster shows the score for a set of competing policies; a higher score is better for the agent.

To evaluate test phase performances, we conducted round-robin tournaments in which the participants were trained agents. Table 1 shows the round-robin tournament results for the four scenarios. The trained models ran 100 episodes with 50 random seeds. The boldface represents the highest reward for each corresponding scenario. Fig 5 shows an average performance plot to facilitate the interpretation of these comparative results. In this plot, we can measure the robustness of performance when encountering different opponents because each bar indicates the average rewards of various competitors. In all cases, the CTRL is improved by the AMLAPN, and the AMLAPN(adv) shows similar or better performances than the AMLAPN(own). Moreover, the proposed methods outperformed the MADDPG in most cases. In some cases, although the MADDPG shows the highest relative scores, we can identify the effectiveness of the AMLAPN in constantly improving the performance of the CTRL. Thus, we can conclude that the AMLAPN is also helpful for learning robust policies.’

**Table 1:** Round-robin tournament results for the four scenarios. Standard deviations are shown within parentheses.

Scenario	Method		Reward			
	Team A	Team B	Team A		Team B	
Physical deception	MADDPG	MADDPG	4.47	(18.74)	6.71	(2.09)
		w/o AMLAPN	-1.06	(22.48)	9.42	(3.55)
		AMLAPN(own)	-10.93	(20.33)	14.38	(6.36)
		AMLAPN(adv)	-23.78	(14.48)	21.06	(5.42)
	w/o AMLAPN	MADDPG	119.90	(17.52)	7.57	(2.34)
		w/o AMLAPN	105.15	(18.29)	16.04	(10.36)
		AMLAPN(own)	94.44	(11.71)	20.73	(11.09)
		AMLAPN(adv)	73.78	(13.9)	**32.37**	(8.68)
	AMLAPN(own)	MADDPG	**153.06**	(6.81)	6.44	(2.11)
		w/o AMLAPN	143.16	(8.92)	11.78	(2.96)
		AMLAPN(own)	122.31	(14.23)	22.49	(7.68)
		AMLAPN(adv)	107.44	(17.71)	29.88	(9.42)
	AMLAPN(adv)	MADDPG	144.44	(12.15)	6.28	(2.02)
		w/o AMLAPN	139.91	(13.34)	9.16	(2.86)
		AMLAPN(own)	130.21	(12.04)	14.04	(3.99)
		AMLAPN(adv)	102.12	(15.25)	28.25	(7.14)
Keep away	MADDPG	MADDPG	-7.49	(1.02)	-9.11	(3.17)
		w/o AMLAPN	-7.65	(0.99)	-1.93	(2.5)
		AMLAPN(own)	-7.77	(0.93)	-0.97	(0.85)
		AMLAPN(adv)	-7.80	(0.93)	-0.87	(0.82)
	w/o AMLAPN	MADDPG	-8.56	(5.6)	-8.66	(2.97)
		w/o AMLAPN	-8.78	(5.54)	-0.71	(3.46)
		AMLAPN(own)	-8.88	(5.5)	-0.60	(3.05)
		AMLAPN(adv)	-8.90	(5.48)	**-0.52**	(2.87)
	AMLAPN(own)	MADDPG	**-6.51**	(0.38)	-9.97	(2.94)
		w/o AMLAPN	-6.71	(0.42)	-2.82	(2.38)
		AMLAPN(own)	-6.84	(0.37)	-1.84	(0.37)
		AMLAPN(adv)	-6.87	(0.37)	-1.72	(0.38)
	AMLAPN(adv)	MADDPG	-6.53	(0.36)	-9.94	(2.9)
		w/o AMLAPN	-6.70	(0.41)	-2.85	(2.36)
		AMLAPN(own)	-6.85	(0.37)	-1.86	(0.36)
		AMLAPN(adv)	-6.85	(0.37)	-1.75	(0.38)
Predator-prey	MADDPG	MADDPG	**-7.96**	(1.4)	23.89	(4.2)
		w/o AMLAPN	-8.80	(4.51)	26.39	(13.52)
		AMLAPN(own)	-10.88	(2)	32.65	(6)
		AMLAPN(adv)	-12.96	(2.05)	38.88	(6.16)
	w/o AMLAPN	MADDPG	-17.60	(9.57)	52.81	(28.7)
		w/o AMLAPN	-15.80	(5.65)	47.40	(16.94)
		AMLAPN(own)	-24.66	(15.82)	73.98	(47.46)
		AMLAPN(adv)	-28.21	(15.95)	**84.63**	(47.86)
	AMLAPN(own)	MADDPG	-15.19	(3)	45.56	(9.01)
		w/o AMLAPN	-17.87	(8.27)	53.61	(24.8)
		AMLAPN(own)	-19.56	(3.44)	58.68	(10.31)
		AMLAPN(adv)	-25.53	(4.53)	76.59	(13.58)
	AMLAPN(adv)	MADDPG	-15.06	(1.73)	45.19	(5.2)
		w/o AMLAPN	-17.08	(7.95)	51.24	(23.84)
		AMLAPN(own)	-19.31	(3.81)	57.93	(11.43)
		AMLAPN(adv)	-25.02	(2.66)	75.06	(7.99)
Complex predator-prey	MADDPG	MADDPG	-29.37	(5.66)	121.20	(22.69)
		w/o AMLAPN	-7.93	(6.58)	35.64	(25.6)
		AMLAPN(own)	-14.99	(2.62)	63.70	(10.34)
		AMLAPN(adv)	-15.17	(3.81)	64.39	(15.4)
	w/o AMLAPN	MADDPG	-33.59	(9.99)	**138.96**	(40.09)
		w/o AMLAPN	-6.55	(4.45)	30.70	(17.81)
		AMLAPN(own)	-23.30	(19.02)	97.72	(76.17)
		AMLAPN(adv)	-27.89	(18.58)	115.58	(74.37)
	AMLAPN(own)	MADDPG	-25.77	(5.03)	106.67	(19.75)
		w/o AMLAPN	-5.03	(4.14)	24.18	(16.44)
		AMLAPN(own)	-8.94	(2.71)	39.58	(10.57)
		AMLAPN(adv)	-9.88	(3.09)	43.38	(12.34)
	AMLAPN(adv)	MADDPG	-25.32	(5.9)	104.43	(23.86)
		w/o AMLAPN	**-4.67**	(2.34)	22.28	(9.51)
		AMLAPN(own)	-5.77	(2.31)	26.30	(9.44)
		AMLAPN(adv)	-10.70	(4.48)	46.20	(18.24)

### The effects of approximate model learning

In this section, we evaluated the approximate model learning under partial observations. To do this, we modified two scenarios: the predator-prey and the complex predator-prey. The number of predators and prey were increased to six and eight in the predator-prey scenario and the complex predator-prey scenario, respectively. In addition, there were the same number of predators and prey in these settings. The most crucial modifications are that agents cannot observe the positions of teammates and that all positions represent their absolute coordinates. Under these settings, many reinforcement learning algorithms suffer from agents observing only a part of the environment (partial observation). To overcome this limitation, agents can infer hidden information by exploiting only observed information. The experiments were conducted to examine whether the approximate model learning helped agents exploit the information they observed.

**Fig 6:**
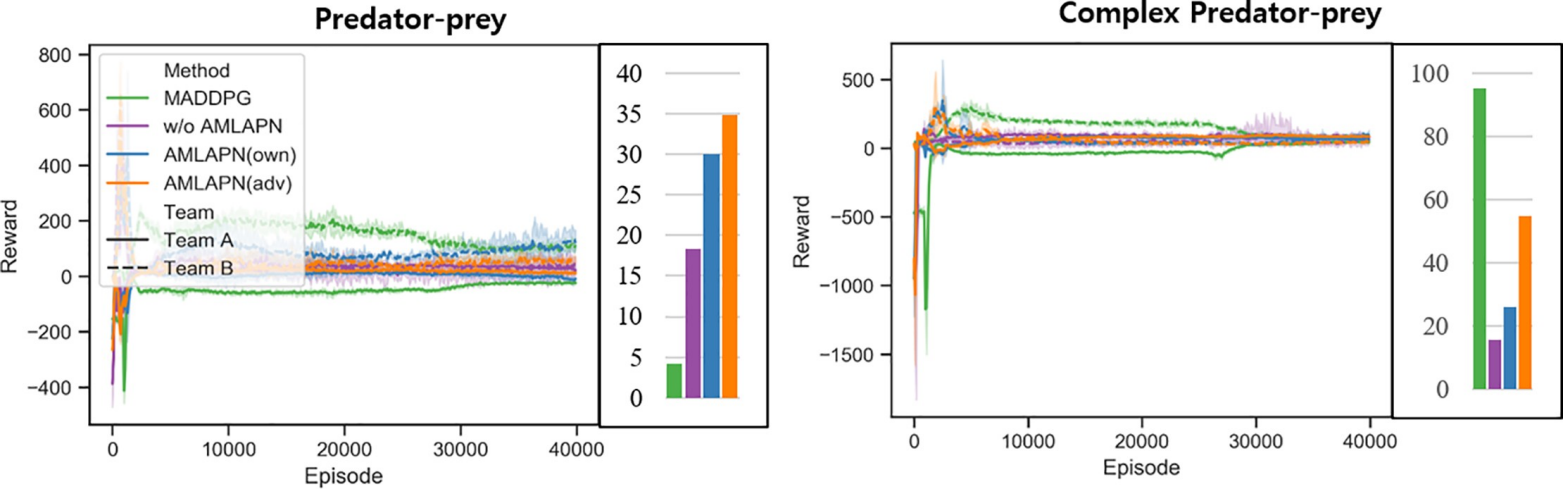
**Learning curves in the partial observation environments:** episode rewards in predator-prey (*left*) and episode rewards in complex predator-prey (*right*) scenarios. Each bar cluster represents the converged episode reward at the end of training. The shading region is a 95% confidence interval across the different random seeds.

Fig 6 shows the learning curves and convergence points of the rewards sum of two teams under partial observation conditions. Unlike in previous experimental results, the AMLAPN(own) shows higher performance than the other methods in all scenarios. This result implies that approximate model learning for inferring one’s state transition is more important than opponent modeling. In the case of the AMLAPN(adv), the inference capabilities of agents’ own state transition could be degraded by opponent modeling because the actor and critic networks simultaneously minimize losses for learning environment dynamics and opponent behavior. Thus, we can conclude that environment dynamics are more important than opponent modeling when agents can access partial observations, even in competitive tasks.

**Fig 7:**
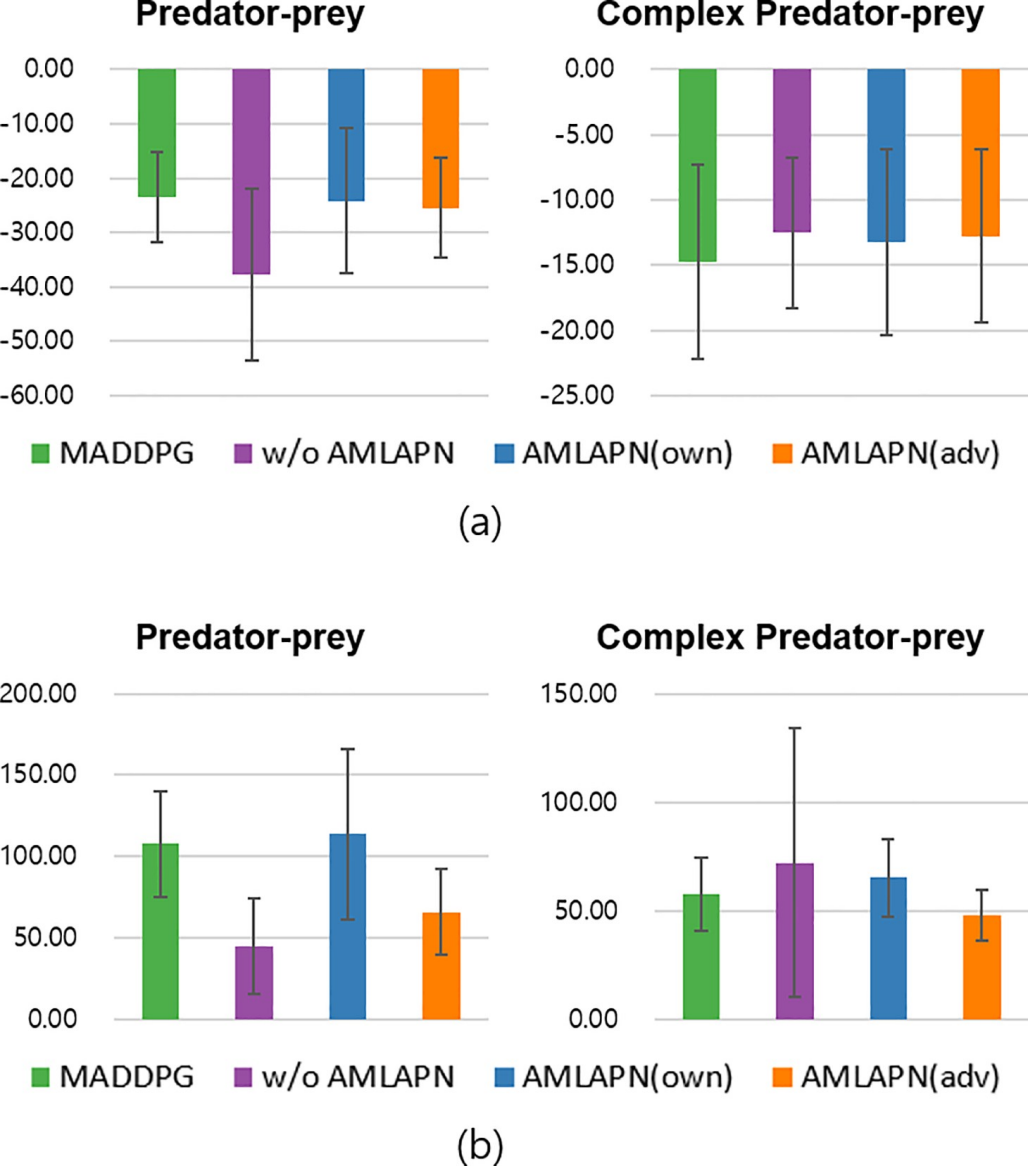
Average relative performances with partial observation in round-robin tournament evaluations: performances of team A trained by four methods (a) and performances of team B trained by four methods (b). Each bar cluster shows the score for a set of competing policies; a higher score is better for the agent.

As with previous experiments, we also conducted round-robin tournaments to evaluate test phase performances. The participants were trained the four different methods. Table 2 shows the round-robin tournament results for the four scenarios with partial observations. The trained models ran a hundred episodes with ten random seeds. The boldface represents the highest rewards for each corresponding scenario. Fig 7 shows an average performance plot to facilitate the interpretation of these comparative results. In the predator-prey scenario, the AMLAPN(own) shows the highest performance against other methods, indicating that the AMLAPN perceiving environment dynamics is important for obtaining robust policies against various opponents. However, in the case of the complex predator-prey scenario, the AMLAPN(adv) and CTRL without AMLAPN earned the highest average rewards on team A and team B, respectively. Moreover, the AMLAPN does not improve team B’s performance in solving the task in the complex predator-prey scenario. To solve this task, especially, team B (predators) must cooperate based on observation of the leader agent. In this case, the AMLAPN is hard to improve multiple agents with different roles because of the limited information about adversary’s observation. This implies that there is room for improvement of the proposed approximate model learning under complex environments.

**Table 2:** Round-robin tournament results for the four scenarios with partial observations. Standard deviations are shown within parentheses.

Scenario	Method		Reward			
	Team A	Team B	Team A		Team B	
Predator-prey	MADDPG	MADDPG	-25.25	(4.57)	75.74	(13.72)
		w/o AMLAPN	-13.92	(6.47)	41.76	(19.41)
		AMLAPN(own)	-34.12	(17.64)	102.37	(52.93)
		AMLAPN(adv)	-20.37	(4.58)	61.12	(13.75)
	w/o AMLAPN	MADDPG	-49.98	(14.99)	149.93	(44.98)
		w/o AMLAPN	-16.78	(10.65)	50.34	(31.95)
		AMLAPN(own)	-54.02	(21.07)	**162.06**	(63.2)
		AMLAPN(adv)	-29.90	(16.44)	89.70	(49.32)
	AMLAPN(own)	MADDPG	-33.27	(14.75)	99.82	(44.24)
		w/o AMLAPN	**-14.24**	(13.42)	42.72	(40.27)
		AMLAPN(own)	-27.82	(13.63)	83.46	(40.88)
		AMLAPN(adv)	-21.28	(11.5)	63.84	(34.5)
	AMLAPN(adv)	MADDPG	-35.02	(8.53)	105.07	(25.59)
		w/o AMLAPN	-14.76	(8.78)	44.28	(26.33)
		AMLAPN(own)	-35.54	(17.3)	106.62	(51.91)
		AMLAPN(adv)	-16.42	(2.66)	49.26	(7.97)
Complex predator-prey	MADDPG	MADDPG	-13.64	(3.73)	62.04	(15.01)
		w/o AMLAPN	-18.18	(18.76)	80.98	(74.46)
		AMLAPN(own)	-15.81	(4.71)	71.57	(18.2)
		AMLAPN(adv)	-11.34	(2.48)	53.01	(8.94)
	w/o AMLAPN	MADDPG	-18.40	(8.57)	81.32	(33.82)
		w/o AMLAPN	-8.98	(7.94)	44.32	(31.94)
		AMLAPN(own)	-11.30	(2.59)	53.56	(11.45)
		AMLAPN(adv)	-11.42	(4.13)	53.04	(16.57)
	AMLAPN(own)	MADDPG	**-8.65**	(2.22)	41.11	(8.51)
		w/o AMLAPN	-20.54	(20.14)	**89.64**	(81)
		AMLAPN(own)	-14.87	(3.27)	66.56	(14.13)
		AMLAPN(adv)	-8.98	(2.83)	42.96	(11.43)
	AMLAPN(adv)	MADDPG	-9.85	(2.37)	46.22	(9.47)
		w/o AMLAPN	-16.66	(15.07)	73.36	(59.62)
		AMLAPN(own)	-15.56	(6.75)	69.08	(28.17)
		AMLAPN(adv)	-9.04	(2.49)	42.56	(9.86)

According to the above results in the simulation environments, the following can be obtained: (1) the proposed AMLAPN promotes the learning process of CTRL for understanding environment dynamics, including opponent behavior, and (2) the proposed AMLAPN without opponent modeling shows relatively better performance in the case of partially observed states.

## Conclusions

In this study, we examined the problem of learning multi-agent policies in competitive environments in which agents receive global reward sharing with their team. In this setting, learning algorithms must cope with the following challenges: (1) partial observability, (2) credit assignment problem of the global reward, (3) performance degradation for learning algorithms because of nonstationary and non-Markovian environments, and (4) evolving opponent policies under imperfect information game conditions. We propose the CTRL with approximate model learning to tackle these issues in multi-agent environments based on centralized training for each team. Through deterministic policy gradient methods and recurrent actor–critic networks, we demonstrate the superiority of the proposed method over another multi-agent reinforcement learning algorithm, the MADDPG, which is a representative method. In particular, we identify that the AMLAPN improves the performance of the CTRL by promoting an understanding of the environment dynamics and opponent behavior. Our experiments with four competitive multi-agent environments demonstrate the superiority of the proposed method compared to competitors in terms of learning efficiency. Moreover, we reveal that the proposed method produces robust performances against the MADDPG by performing round-robin tournament evaluations.

There are a couple of interesting directions for future research. One is to consider the dependency between opponent policies and state transition in auxiliary prediction networks for improving performance in complex environments. Another interesting direction is to use the transformer [35], an alternative recurrent neural network, in the actor–critic architecture to handle the sequencing of agents with no arbitrary orders.
